# 3D DEM Simulations and Experiments on Spherical Impactor Penetrating into the Elongated Particles

**DOI:** 10.3390/ma16041664

**Published:** 2023-02-16

**Authors:** Ping Li, Yanjie Li, Xia Hua, Yu Guo, Jennifer Sinclair Curtis

**Affiliations:** 1 School of Technology, Beijing Forestry University, Beijing 100083, China; 2 College of Mechanical Engineering, Zhejiang University of Technology, Hangzhou 310023, China; 3 Department of Engineering Mechanics, Zhejiang University, Hangzhou 310027, China; 4 College of Engineering, University of California, Davis, One Shields Ave, Davis, CA 95616, USA

**Keywords:** impaction, elongated particles, impact experiment, discrete element method, particle orientation

## Abstract

In this study, a brass or glass spherical impactor vertically penetrating into a granular bed composed of mono-sized spherical or elongated particles was simulated with three-dimensional (3D) discrete element method (DEM). Good agreement of the particle masses in the cup before and after penetration can be found in the simulations and experiments. The effects of particle length (*L*_p_), friction coefficient, and particle configuration on the penetration depth of the impactor, ejecta mass, and solid volume fraction describing the response of the granular bed are discussed. The penetration depth is negatively correlated with *L*_p_ as the corresponding solid volume fraction of the granular bed decreases. A smaller friction coefficient leads to a larger penetration depth of the impactor and more ejection of particles. When the impactor is penetrating the *L*_p_ = 10 mm elongated particles, the penetration depth is negatively correlated to the order parameter and solid volume fraction.

## 1. Introduction

Non-spherical particles are widely ubiquitous in nature and industry; for instance, micro-particles of various shapes have been applied in drug transportation and mixing. Particle shape has been shown to influence various processes including drug transport and crystal preparation [1,2,3,4]. When non-spherical elongated particles are packed, the internal microstructure of the granular bed has a complex and important influence on the macroscopic mechanical response of the material, which is different from that of spherical particles. Furthermore, the mechanical behavior of non-spherical particles affected by the impact will have a significant influence on transportation and industrial production. Therefore, the study of the impact into elongated particles is of great importance. 

The finite element method (FEM) is an effective numerical method for continuum material, such as variational phase-field problems [5,6], quasistatic frictional contact problems [7], and elastic systems [8]. In addition, the discrete element method (DEM) is one of the most effective numerical methods for studying the dynamic properties of granular medium; it is a discontinuous numerical method first proposed by Cundall in 1971 [9]. Based on the theory of Newton’s second law and the contact model between particles, the DEM can be applied to the particle dynamics and kinematics analysis by calculating the displacement and force of each particle in the granular system based on the explicit time step iteration.

Both 2D [10,11,12,13,14] and 3D [15,16,17] discrete element models have been used in numerical simulations of object impacts into target granular medium. Studies have shown that the response of the granular bed and impactor depends on many factors, such as the shape, size, angle, and penetration velocity of the impactor [10,18,19,20,21], and the friction coefficient and porosity of the granular bed [11]. In addition, the energy dissipates drastically during the transient impact process, which has a complex relationship with the physical parameters or material properties of the granular medium [22]. Based on studies of spherical particles, the effect of elongated particles can be discussed comparatively.

Numerical models of regular non-spherical particles such as cylinders [23], sphero-cylinders [24,25], polyhedrons [26,27], super-ellipsoids [28,29], multi-super-ellipsoids [30,31], and arbitrarily shaped elements [32] have been developed gradually. The packing of elongated particles was discussed by changing various parameters, such as coefficient of friction and coefficient of restitution [33]. The behavior of elongated particles with varying lengths was explored by discharging them from a rectangular hopper [34]. Studies have shown that the length of the particle can be used to adjust the buffer capacity for the non-spherical particle system [35]. Besides, the effects of particle length and configuration of granular bed on impact have not been discussed, and the effect mechanisms of the elongated particle system have not been studied comprehensively. This study can provide a fundamental insight for the mechanical behavior of impact into non-spherical particles.

In this study, we carried out experiments on vertical impact into granular material and compared the ejecta masses and initial solid volume fractions of the granular bed between experiments and simulations, considering spherical and elongated particles as the objective granular medium. For the simulations based on DEM, we investigated the behaviors of the ejected particles, the particles in the cup, and the impactor. For the above three objects of study, we considered the effects of some key factors according to their different behavior characteristics, including the length of elongated particles, the friction coefficient, the shape of the granular cup, and the configuration of particles.

## 2. Numerical Model and Methodology

In this study, the translational and rotational movements of particles are governed by Newton’s second law of motion. The equations are written as follows:

(1) $$F_{i}=m_{i}\frac{dv_{i}}{dt}$$

(2) $$T_{i}=I_{i}\frac{d\omega_{i}}{dt}-(I_{i}\cdot \omega_{i})\times \omega_{i}$$

in which $m_{i}$, $v_{i}$, $I_{i}$, and $\omega_{i}$ are the mass, translational velocity, moment of inertia, and rotational velocity of particle *i*, respectively, and ***F****_i_* and ***T****_i_* are external forces and torques exerting on it.

The contact types of the sphero-cylindrical particle for the DEM simulation are classified into four groups according to the normal contact forces, as shown in Table 1. The normal contact force calculation and implemented contact detection in this study were proposed by Kidokoro et al. [25] and modified by Guo et al. [36]. The magnitudes and directions of normal and tangential contact forces are determined by contact position and the overlap of the two contacting particles. The tangential force model is the Mindlin model, which only takes into account the static process in this study.

For the normal and tangential damping force [36], $F_{n}^{d}$ and $F_{t}^{d}$ are determined by

(8) $$F_{n}^{d}=-c\beta \sqrt{2S_{n}m^{*}}v_{n}$$

(9) $$F_{t}^{d}=-\beta \sqrt{2S_{t}m^{*}}v_{t}$$

where *c* is equal to 1 if the normal contact force *F*_n_ is proportional to $\delta_{n}$, and *c* is equal to $\sqrt{\frac{5}{6}}$ if the normal contact force *F*_n_ is proportional to $\delta_{n}{}^{\frac{3}{2}}$. m^*^ is equivalent mass, $m^{*}={\left(\frac{1}{m_{1}}+\frac{1}{m_{2}}\right)}^{-1}$, and *m*_1_ and *m*_2_ are the masses of two contacting particles. *v*_n_ and *v*_t_ are the normal and tangential components of relative velocity, respectively. $\beta =\frac{-\mathrm{lne}}{\sqrt{\pi^{2}{+(\mathrm{lne})}^{2}}}$ is the contact damping coefficient, where *e* is the coefficient of restitution. *S*_n_ is normal contact stiffness, and *S*_t_ is tangential contact stiffness, given by $S_{n}=\frac{dF_{n}}{d\delta_{n}}$ and $S_{t}={8G}^{*}a$, respectively.

## 3. Experimental and Numerical Setup

**Figure 1:**
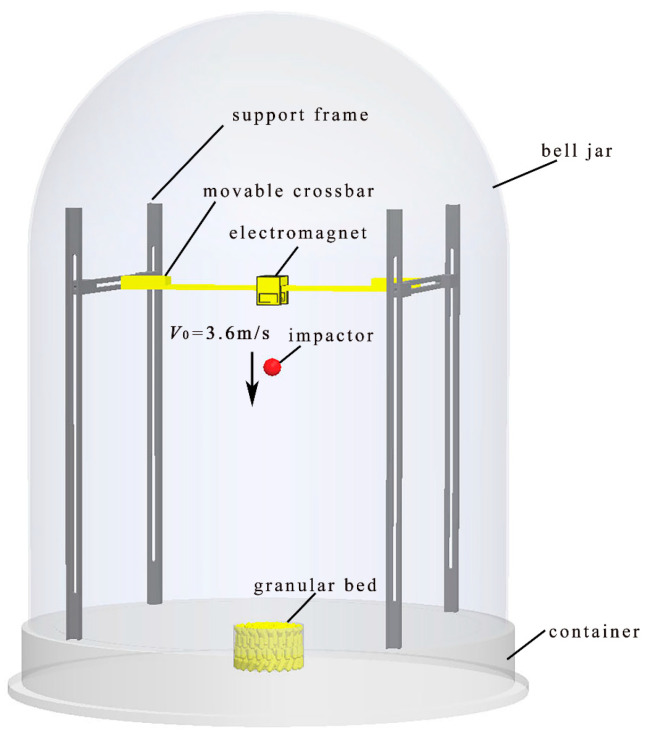
Schematic drawing of the experimental apparatus. (The arrow represents the velocity direction of the impactor.)

The experimental apparatus used in this study is the same as what we used in the previous study [17]. Figure 1 shows the schematic of the experimental apparatus. The experiment was carried out in an airtight vacuum chamber (<200 mTorr), taking no account of the gas drag on the particles during the impact process. The impactor has a steel dot pasted on the top. Firstly, the impactor was fixed by an electromagnet at the central position of the crossbar at a height of *H* = 661 mm above the top of the granular bed. Then, the cylindrical particles were poured into the cup, and the surface of the granular bed was smoothed with a scraper. The total mass of the cup and the particles in it, *m*_0_, was weighed. The air was then pumped out until the pressure in the chamber was lower than 200 mTorr. Finally, the impactor was released from static state under gravity, and the vertical velocity of the impactor was calculated by $V_{0}=\sqrt{2gH}$ (where g is the acceleration of gravity). After all particles reached the stable state, the total mass of the cup and particles remaining in it, *m*, was weighed. The mass of ejected particles can be obtained by the equation Δ*m* = *m*_0_ − *m*. The objective particles used in the experiments are the steel balls and steel cords cut into different lengths (*L*_p_ = 4 mm and 6 mm), whose diameters are 2 mm. The impactors are brass and glass spheres, whose impact velocities are 3.60 m/s. The parameters of all the materials used in experiment are shown in Table 2.

**Figure 2:**
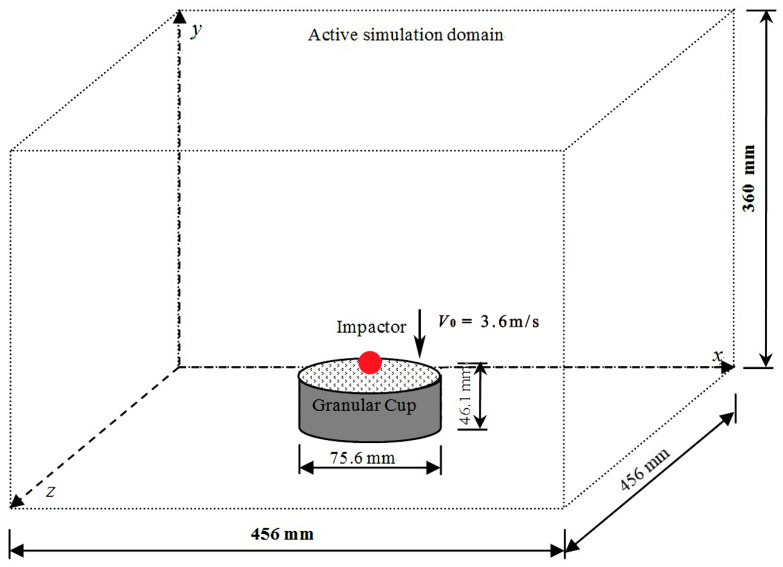
Schematic diagram of numerical setup. (The arrow represents the velocity direction of the impactor.)

The setup of DEM simulations is shown in Figure 2. The geometric and physical parameters of the cup, granular particles, and impactors are chosen from the experimental study. We mainly focus on the particles in the cup and the initial trajectory of ejected particles, and the cuboidal active simulation domain is big enough and will not affect the motion of ejected particles. The implementation of DEM simulation in this study was as follows: Firstly, the computing domain was divided into same-sized cuboid cells, and a certain number of particles were generated without contact and mapped into the cells. The particles were generated within the cylindrical domain with the same diameter as the cup and enough height. The generated particles then fell downward under gravity and packed in the cup until the cup was filled up with the particles. The translational and rotational motions of particles were controlled by Newton’s second law of motion. The normal and tangential contact forces were calculated by the overlap between two contacting objects according to various contact models. When all particles settled down, the impactor was then released with an initial vertical velocity of 3.60 m/s right above the particle bed and penetrated into the particle bed in the center.

In the DEM simulations, the particles are steel spheres and elongated steel particles of *L*_p_ = 4 mm, 6 mm, 8 mm, 10 mm with a hemisphere cap at each end. The diameters of the spheres and elongated particles are equal to 2 mm, which is the same as the cylindrical particles used in experiments. Brass and glass spherical impactors with the same diameter are used in both experiments and simulations. The diameter, the height, the density, the coefficient of friction, and the coefficient of restitution are measured experimentally. Young’s modulus and Poisson’s ratio are selected from a handbook [37]. The physical and geometric parameters of particles and impactors are summarized in Table 2. Contact detection of the elongated particles in this study is the same as the sphero-cylinder model in the literature [24]. The difference is that the elongated sphero-cylinder used here is rigid, and it was a flexible elastic model in the literature.

**Figure 3:**
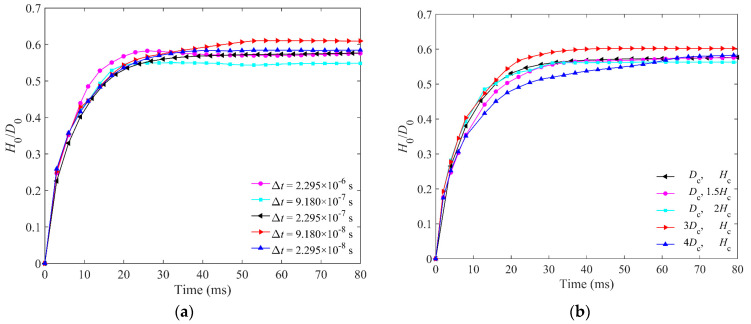
(**a**) Comparison of the penetration depth of glass impactor for five different time steps in DEM simulations. (**b**) Comparison of the penetration depth of glass impactor for five different cups in DEM simulations. (The length of elongated particles is 10 mm, the velocity of glass impactor is 3.60 m/s, and *D*_c_ and *H*_c_ are shown in Table 2).

The time step and boundary effect are two important parameters for DEM simulations; therefore, the sensitivities of these two parameters are studied before we determine the final values of them. The penetration depths of five different time steps are compared. The *L*_p_ = 10 mm steel elongated particles and the glass impactor are chosen for simulation, and the parameters of materials are shown in Table 2. *H*_0_ and *D*_0_ are the penetration depth and diameter of the impactor, respectively. *H*_c_ and *D*_c_ are the height and diameter of the cup, respectively. Figure 3a shows the normalized penetration depth of the glass impactor for different time steps, which shows the limited effect of different time steps. It can be found that the final penetration depths of the glass impactor for different cup heights are very close. The radius and height of the cup have little effect on the penetration depth. There is no obvious tendency with the change of time step or cup size. The average penetration depths of different time steps and cup sizes are both approximately 0.58 *D*_0_. The penetration depths of different time steps are in the range of 0.55 to 0.61 *D*_0_, and those of different cups are in the range of 0.56 to 0.60 *D*_0._ Therefore, we chose ∆*t* = 2.295 × 10^−7^ s, and *D*_c_ and *H*_c_ as the final time step and cup size.

## 4. Results and Discussion

In this section, the key parameters on impact are discussed, including particle length, friction coefficient, cup shape, and particle configuration. In addition, three objects will be studied, including the particles out of the cup, the particles in the cup, and the impactor. Their fundamental parameters are analyzed, such as the ejecta mass, the penetration depth, the solid volume fraction, and the kinetic energy.

### 4.1. Effect of Particle Length

The effect of particle length on impact is studied in this section, and spherical particles and elongated particles of four different lengths with the same diameter are discussed. Two materials of brass and glass impactor with impact velocities of 3.60 m/s are used. The friction coefficients are the same as the base case and material parameters are shown in Table 2.

The kinetic energy of the ejected particles, the impactor, and the particles left in the cup are calculated. The kinetic energy includes rotational kinetic energy and translational kinetic energy. For particle *i* with mass *m_i_* and moment of inertia *I_i_*, the translational velocity and rotational velocity at time *t* are defined as *v_i_*(*t*) and *w_i_*(*t*), respectively, and the total kinetic energy of *N* particles is given by

(10) $$E_{k}\left(t\right)=\sum_{i}^{N}\frac{1}{2}m_{i}v_{i}^{2}\left(t\right)+\sum_{i}^{N}\frac{1}{2}I_{i}\omega_{i}^{2}\left(t\right)$$

The initial kinetic energy of the impactor is $E_{k0}=\frac{1}{2}MV_{0}{}^{2}$, and the initial potential energy of the impactor is $E_{p0}=MgH_{0}$ (the position of zero gravitational potential energy is at the final stopping point of the impactor). The mass of impactor *M* is calculated by the density and size shown in Table 2. We take the percentage of the energy to the sum of *E*_k0_ and *E*_p0_ as the scaled energy.

#### 4.1.1. The Particles out of the Cup

**Figure 4:**
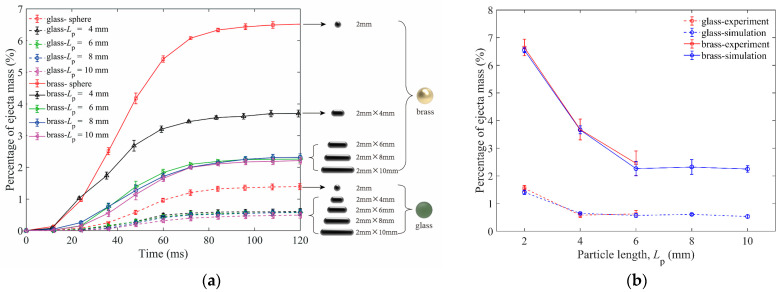
(**a**) Influence of particle length on ejecta masses for both glass and brass impactors in DEM simulations. (**b**) Comparison of ejecta mass as a function of particle length between experiments and simulations. (The length of 2 mm represents the data of 2 mm spheres here and all the simulation parameters are from Table 2).

The ejecta mass and the kinetic energy of ejected particles $E_{k}^{e}$ are shown in this section. Figure 4a,b show the ejecta masses for granular beds composed of spheres and elongated particles of different lengths with the same diameter in simulations and experiments.

Figure 4a is the dynamic change of ejecta mass and Figure 4b is the comparison of the final ejecta masses between simulations and experiments. In Figure 4a, we set the time that the impactor touches the top of the particle bed as *t* = 0 ms. The end time of particle ejection for brass and glass impactors is about 90 ms. It is obvious that the ejecta masses of the brass impactor are larger than those of the glass impactor due to higher impactor energy. For the brass impactor, the ejecta masses of *L*_p_ = 6 mm, 8 mm, and 10 mm elongated particles are closer and smaller than that of *L*_p_ = 4 mm. For the glass impactor, the ejecta masses of *L*_p_ = 4 mm, 6 mm, 8 mm, and 10 mm elongated particles are approximately equal and smaller than that of 2 mm spheres.

In Figure 4b, the agreement of solid volume fractions and ejecta masses between experiments and simulations can be found. The results show that the ejecta mass of spherical particles is much larger than that of elongated particles. In this simulation, the method of changing the aspect ratio is increasing the length of particles (keeping the diameter unchanged). In this way, the mass of a single particle also increases as a result of increasing particle length. When the length of particles increases to 6 mm, the impact energy of the glass impactor cannot make more elongated particles eject from the cup because of the large mass of a single particle. Therefore, the percentage of total ejecta mass of 6 mm particles is close to that of 4 mm, and their values are only 0.5%, indicating that the total mass of ejected particles is very small. Similarly, when the length of particles increases to 8 mm, the ejecta mass for the brass impactor is almost the same as that of 6 mm. By comparing the results of the two impactors, it can be concluded that when the mass of the elongated particle increases to a certain value, the transferred energy cannot support the ejection of more particles.

**Figure 5:**
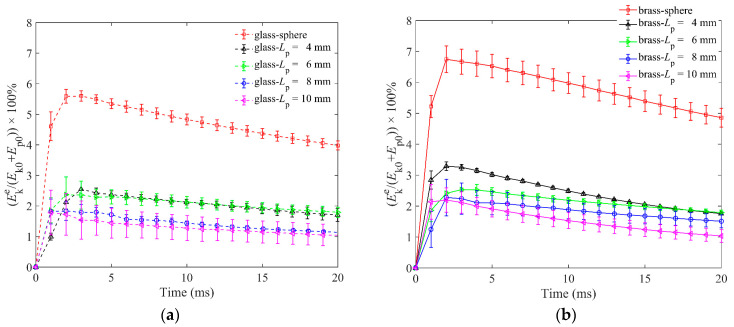
Kinetic energy $E_{k}^{e}$ of ejected particles as a function of time for granular bed of spherical particles and elongated particles of four particle lengths in DEM simulations: (**a**) is for the glass impactor, and (**b**) is for the brass impactor.

Figure 5a,b show the kinetic energy of ejected particles for two impactors. The $E_{k}^{e}$ reached its peak value at about 2 ms when the particles collided with the impactor and gained the maximum velocities. Some particles began to launch upwards and fly in the air along parabolic trajectories. The ejected particles were moving upward along the parabolic trajectory before 20 ms, and their velocities were hence decreasing, resulting in the decreasing kinetic energy. It is easy to find that the peak values of $E_{k}^{e}$s for spherical particles are much larger than those of elongated particles for both glass and brass impactors because spherical particles roll more easily than elongated ones. Comparatively, the $E_{k}^{e}$s of different elongated particles lengths are obviously smaller than those of spherical particles, and $E_{k}^{e}$s are slightly smaller as the particles become longer, whether for the glass or brass impactor. We can suppose that the kinetic energy transferred to the ejected particles is to support their movement and more energy will be needed to drive a particle moving if its mass becomes larger. In this study, we changed the aspect ratio of a particle by lengthening the particle and kept its diameter unchanged. Therefore, the $E_{k}^{e}$s of longer particles (with larger masses) are smaller.

#### 4.1.2. The Impactor

The responses of the impactor are investigated by DEM simulations here, including the penetration depth of the impactor (*H*_0_) and the energy of the impactor ($E_{k}^{i}$).

**Figure 6:**
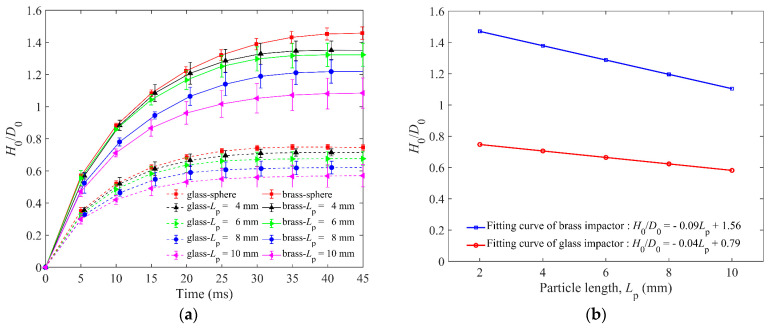
(**a**) *H*_0_/*D*_0_ versus time for granular bed of spheres and elongated particles of four lengths. (**b**) The fitting curves of the *H*_0_/*D*_0_ and particle length *L*_p_ for glass and brass impactors. *H*_0_ and *D*_0_ represent the penetration depth and diameter of the impactor, respectively.

Figure 6 shows the normalized penetration depth of granular beds for spheres and elongated particles. Comparing the *H*_0_/*D*_0_ of brass and glass impactors in Figure 6a,b, it can be found that the final *H*_0_ is negatively correlated to the particle length of the granular bed. For the brass impactor, the largest *H*_0_ is about 1.5 *D*_0_ for the granular bed made of spheres, and the granular beds with *L*_p_ = 10 mm elongated particles have the smallest *H*_0_ of about 1.1 *D*_0_. The shape of the *H*_0_(*t*)/*D*_0_ function of the glass impactor is similar to that of the brass impactor, but its value is less than that of the brass impactor. In addition, the glass impactor stopped earlier than the brass impactor, indicating that the brass impactor with larger kinetic energy took more time to halt the penetration. The time of static state of the glass impactor is earlier than that of the brass impactor, and the static state is defined as the unchanged value of *H*_0_ of the impactor.

**Figure 7:**
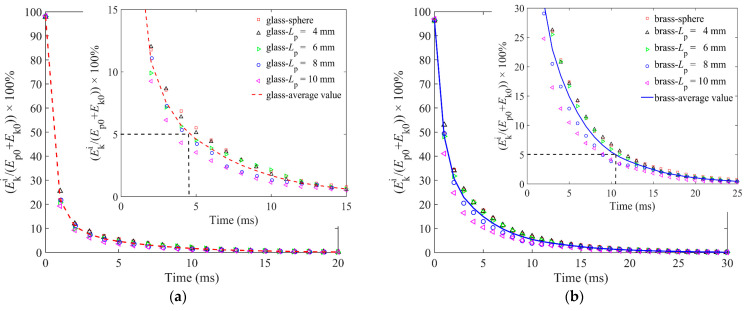
Kinetic energy of impactor $E_{k}^{i}$ as a function of time for granular bed of spherical particles and elongated particles of four lengths: (**a**) is for the glass impactor, and (**b**) is for the brass impactor.

Because of the different penetration depths of the impactor, the initial potential energy is different, resulting in a slight difference of the *E*_k0_ between the glass impactor and brass impactor. As shown in Figure 7a,b, the *E*_k0_ for the glass and brass impactors at *t* = 0 ms are 98% and 96.5%, respectively, indicating that the initial total energy of the impactors dominated by *E*_k0_ and its *E*_p0_ is very small. It can also be found from Figure 7a,b that the kinetic energy of the impactor $E_{k}^{i}$ decreases rapidly from 0 ms to 5 ms. For both glass and brass impactors, the decrease of $E_{k}^{i}$ slightly increases with the increasing particle length, indicating that the dissipation of $E_{k}^{i}$ is faster with a larger particle length. Furthermore, we quantify the average $E_{k}^{i}$ of five lengths, and it shows that the average $E_{k}^{i}$ obeys an exponential-like dissipation. In addition, the brass impactor dissipates 95% of $E_{k}^{i}$ in more than 10 ms, while the glass impactor dissipates 95% of $E_{k}^{i}$ in less than 5 ms. The main reason is that the initial total energy of the glass impactor is smaller than that of the brass impactor.

#### 4.1.3. The Particles in the Cup

To study the dynamic response of the particles in the cup, the average contact force of the granular bed ($F_{\mathrm{pp}}^{c}$), the solid volume fraction (*ϕ*_p_), the granular temperature (*T*_p_), and the kinetic energy are discussed.

**Figure 8:**
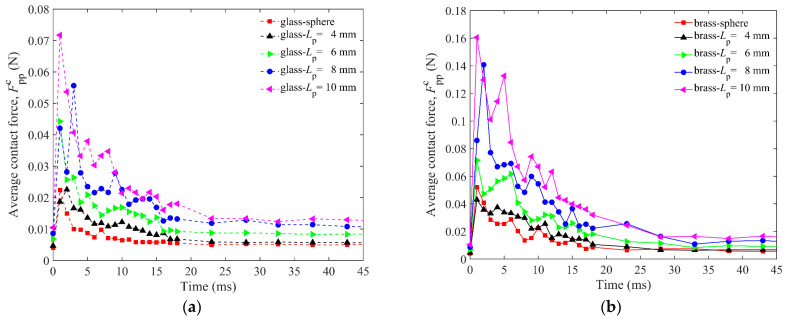
Average contact force of particles $F_{\mathrm{pp}}^{c}$ as a function of time for 2 mm sphere and *L*_p_ = 4 mm, 6 mm, 8 mm, 10 mm elongated particles for DEM simulations: (**a**) is for the glass impactor, and (**b**) is for the brass impactor.

The correlation between *H*_0_ and *L*_p_ can be explained by the average contact force between particles $F_{\mathrm{pp}}^{c}$ in the granular bed. $F_{\mathrm{pp}}^{c}$ is the average resultant force of the normal and tangential forces at all contact points, which can describe the strength to resist the penetration of the impactor, as shown in Figure 8. When the impactor penetrates the granular bed, the particles in the cup move randomly and intensively in the first 10 ms. During this period, the impactor and the particles in the granular bed will be in contact and separated from each other until most of the energy of the impactor is dissipated. In Figure 7, it is easy to find that the energy of the impactor sharply decreases in the first 10 ms. The contact forces show a zig-zagging change, which is a characteristic of the discontinuous medium after impact. The contact force is transmitted through the force chain. The particles are discrete, and the force chain structure in the granular system will change at every moment due to the frequent formation and breakage of the contact forces between particles, which leads to the zig-zagging change of the contact force. Furthermore, a longer particle can easily trigger stronger contact force between particles and has a stronger resistance to the penetration of the impactor, thus reducing the penetration depth of the impactor. This can be used to explain the phenomenon shown in Figure 6.

**Figure 9:**
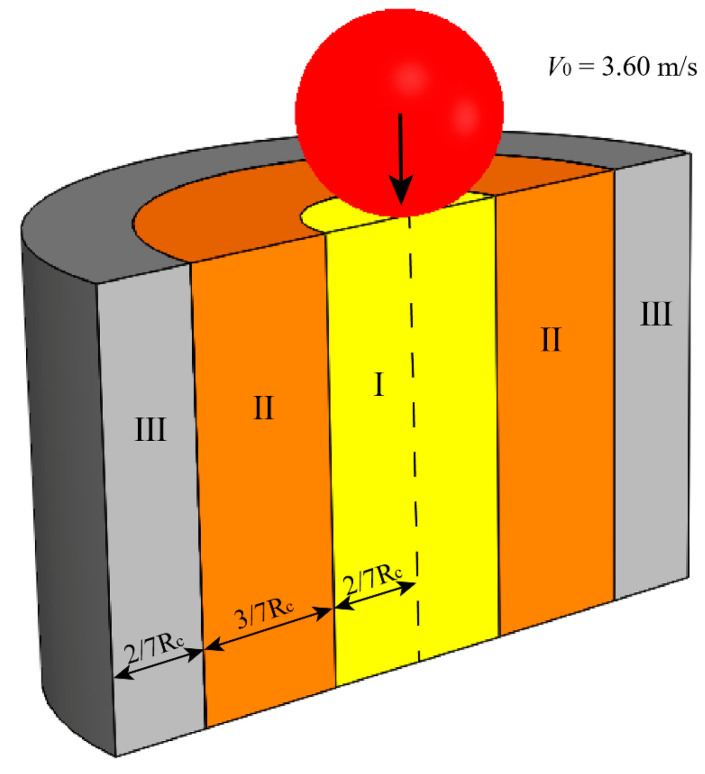
Diagram of sub-region division for granular bed. Region I: 0 < *R*_1_ < 2/7*R*_c_; region II: 2/7*R*_c_ < *R*_2_ < 5/7*R*_c_; and region III: 5/7*R*_c_ < *R*_3_ < *R*_c_. (The particles count in one region if their mass centers fall in this region; *R*_c_ is the radius of the cup).

For the analysis of granular temperature, solid volume fraction, and kinetic energy, the granular bed was divided into three cylindrical regions to investigate the dynamic responses of the particles in the cup, as shown in Figure 9. To distinguish the particles right under the impactor and surrounding the impactor, the radius of region I is chosen to be close to the radius of the impactor. Meanwhile, the thickness of region III cannot be smaller than the length of the longest particle (~2/7*R*_c_). Therefore, the radial widths of region I, II, and III are 2/7*R*_c_, 3/7*R*_c_, and 2/7*R*_c_, respectively. The edge between two adjacent regions is fixed, and particles are free to enter or leave one region.

**Figure 10:**
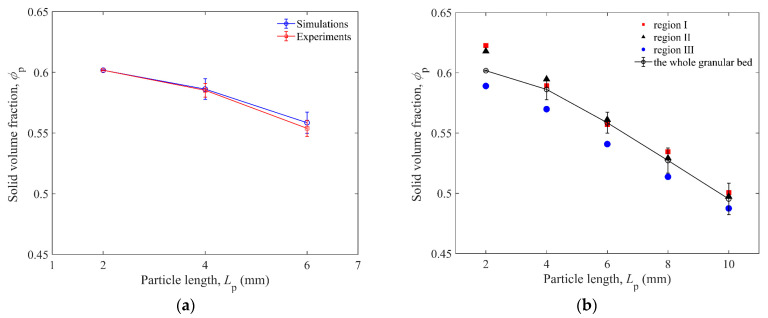
(**a**) Comparison of solid volume fraction *ϕ*_p_ as a function of particle length *L*_p_ between experiments and simulations for both glass and brass impactors. (**b**) Initial solid volume fraction *ϕ*_p_ of the whole granular bed or three regions of granular bed as a function of particle length *L*_p_ before impact. The particle length of 2 mm represents the data of 2 mm spheres here.

Figure 10a shows the comparison of solid volume fractions for granular beds composed of spheres, *L*_p_ = 4 mm, 6 mm elongated particles, impacted by glass and brass impactors in simulations and experiments. The initial solid volume fractions *ϕ*_p_s of the granular beds are compared in Figure 10b. It can be found that the *ϕ*_p_s of region I and II are closer, and those of region III are slightly smaller. It is interesting to find that the *ϕ*_p_ of the granular bed and the *H*_0_ of impactor both decrease with longer particles, which is different from previous studies on spherical particles [13,17]. Due to the random distribution of elongated particles, a 3D cage structure will be formed, and there will be more voids in the structure compared with the granular bed of spheres. Therefore, a smaller *ϕ*_p_ of the bed will be formed by the random packing of longer elongated particles.

**Figure 11:**
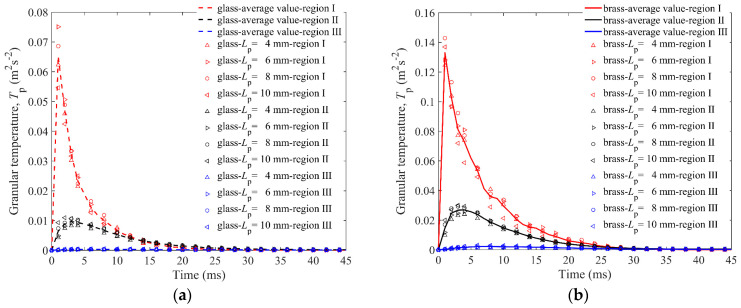
Granular temperature *T*_p_ of particles in three regions versus time for granular bed with *L*_p_ *=* 4 mm, 6 mm, 8 mm, 10 mm elongated particles: (**a**) is for the glass impactor, and (**b**) is for the brass impactor.

Granular temperature [38] *T*_p_ is calculated to describe the fluctuation of translational velocities of the elongated particles in the cup, as shown in Figure 11. The granular temperature of all particles in the cup can be defined as follows:

(11) $$T_{p}={(T}_{p,x}{+T}_{p,y}{+T}_{p,z})/3$$

in which $T_{p,x}={\langle \left(u_{x}-\langle u_{x}\rangle \right)\rangle }^{2}$, $T_{p,y}={\langle \left(u_{y}-\langle u_{y}\rangle \right)\rangle }^{2}$, $T_{p,z}={\langle \left(u_{z}-\langle u_{z}\rangle \right)\rangle }^{2}$, and u−〈u〉 is the fluctuating velocities of the particles. The operator 〈u〉 is used to calculate the average velocity over all particles. The *T*_p_s of three regions are calculated respectively.

In Figure 11, it can be found that the difference between *T*_p_s and *L*_p_s is limited, indicating that *L*_p_ has no significant effect on particle movement. The particle temperature of region I experiences its peak value in the first 1 ms, and the other two regions are about 3 ms and 6 ms, respectively, which shows that the particles move from the middle to the periphery. The peak value of *T*_p_ of the brass impactor is about twice that of the glass impactor in region I and region II. In short, the granular temperature in region I experiences the most dramatic change, followed by region II and region III.

**Figure 12:**
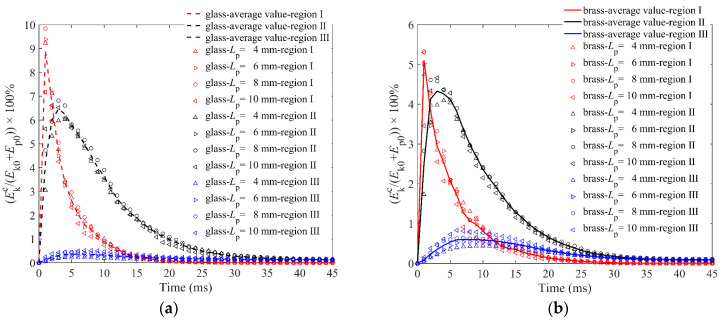
Kinetic energy of particles in the cup $E_{k}^{c}$ in three regions versus time for granular bed with *L*_p_ = 4 mm, 6 mm, 8 mm, 10 mm elongated particles: (**a**) is for the glass impactor, and (**b**) is for the brass impactor.

Figure 12a,b shows the kinetic energy of particles in the cup $E_{k}^{c}$, which is similar to granular temperature. The particle length has little effect on the dissipation of $E_{k}^{c}$ for any region. The particles left in the cup did not move violently but slightly; therefore, the kinetic energy of the particles left in the cup will not be affected by the particle length. In addition, particle length has little effect on the dissipation of $E_{k}^{c}$ for any region. Comparing the energy dissipation of the three regions, it can be found that the $E_{k}^{c}$ of region I has the most drastic change, while that of region III has the smallest change. In addition, the total $E_{k}^{c}$ of three regions of glass impactor is larger than that of brass impactor, which indicates that the impactor with less initial energy transfers its higher energy to the particles in the cup. The brass impactor with higher initial energy transfers more energy to the ejected particles, as shown in Figure 5b.

### 4.2. Effect of Friction Coefficient

**Figure 13:**
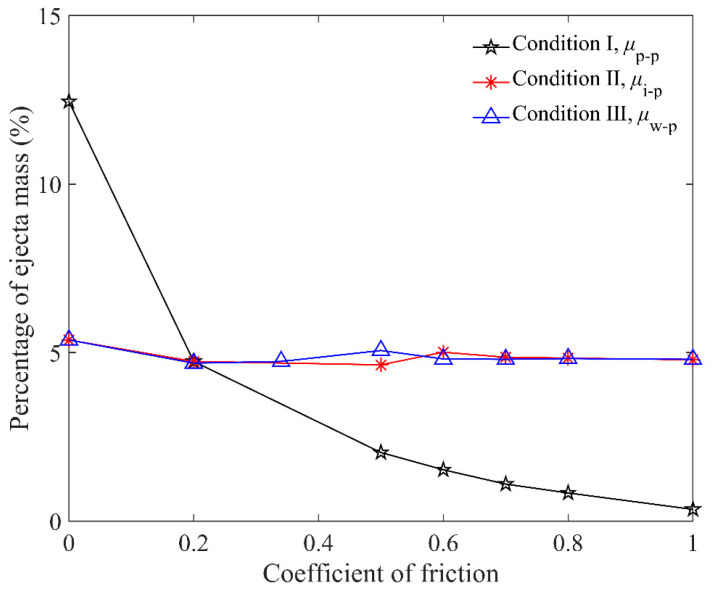
Relationship between percentage of ejecta mass and friction coefficient *μ*_p-p_, *μ*_i-p_, and *μ*_w-p_ for *L*_p_ = 6 mm elongated particles. Condition I: *μ*_i-p_ = 0.2, *μ*_w-p_ = 0.34 and various *μ*_p-p_s; condition II: *μ*_p-p_ = 0.2, *μ*_w-p_ = 0.34 and various *μ*_i-p_s; condition III: *μ*_p-p_ = 0.2, *μ*_i-p_ = 0.2 and various *μ*_w-p_s.

To evaluate the influence of particle friction on penetration, the friction coefficients of the target particles (*μ*_p-p_), impactor and particles (*μ*_i-p_), and cup wall and particles (*μ*_w-p_) were studied. We chose the steel elongated particles of *L*_p_ = 6 mm with initial friction coefficients and the brass impactor for the DEM simulation in this section. Figure 13 shows that the effect of *μ*_p-p_ is dominant. The ejecta mass decreases dramatically when *μ*_p-p_ ranges from 0 to 0.2, and it decreases gently as *μ*_p-p_ changes from 0.2 to 1.0 gradually, while *μ*_i-p_ and *μ*_w-p_ have very little effect on ejecta mass.

**Figure 14:**
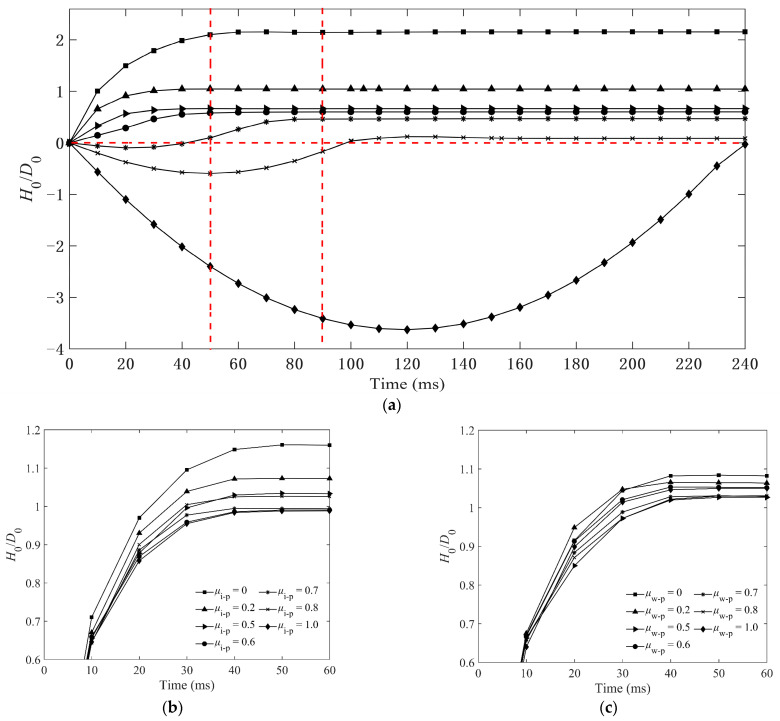
Time evolution of the non-dimensional penetration depth *H*_0_/*D*_0_ of impacting into the granular bed of *L*_p_ = 6 mm particles: (**a**) condition I, (**b**) condition II, and (**c**) condition III. *H*_0_ and *D*_0_ represent the penetration depth and diameter of impactor, respectively. Condition I: *μ*_i-p_ = 0.2, *μ*_w-p_ = 0.34 and various *μ*_p-p_s; condition II: *μ*_p-p_ = 0.2, *μ*_w-p_ = 0.34 and various *μ*_i-p_s; condition III: *μ*_p-p_ = 0.2, *μ*_i-p_ = 0.2 and various *μ*_w-p_s. (The dotted lines are used to clearly mark the position of the impactor at different times.)

**Figure 15:**
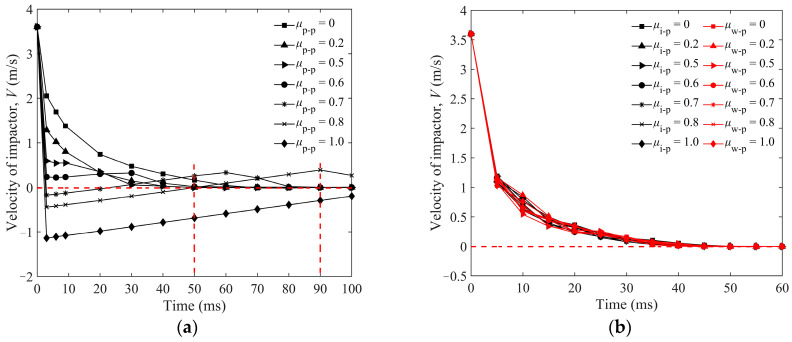
Time evolution of vertical velocity of impactor with sphere impacting into the granular bed of *L*_p_ = 6 mm elongated particles: (**a**) condition I and (**b**) condition II and III. Condition I: *μ*_i-p_ = 0.2, *μ*_w-p_ = 0.34 and various *μ*_p-p_s; condition II: *μ*_p-p_ = 0.2, *μ*_w-p_ = 0.34 and various *μ*_i-p_s; condition III: *μ*_p-p_ = 0.2, *μ*_i-p_ = 0.2 and various *μ*_w-p_s. (The dotted lines are used to clearly mark the velocity of the impactor at different times).

The non-dimensional penetration depth (*H*_0_/*D*_0_) is used to illustrate the correlation among various friction coefficients between particles, which is exhibited in detail in Figure 14a. It is interesting to find that the maximum *H*_0_ of the impactor is about 2*D*_0_ when *μ*_p-p_ = 0 for condition I, and *H*_0_ decreases with increasing *μ*_p-p._ The impactor will rebound to the upside and not penetrate into the granular bed while *μ*_p-p_ ≥ 0.7. However, the friction coefficients of *μ*_i-p_ and *μ*_w-p_ have little effect on *H*_0_. The maximum values of *H*_0_ for different *μ*_i-p_s are less than 0.5 *D*_0_, as shown in Figure 14b, and the *H*_0_ hardly changes for various *μ*_w-p_s in Figure 14c. The vertical velocity of impactor *V* is shown in Figure 15a,b. When *μ*_p-p_ is less than 0.7, the decrease of *V* is positively correlated to *μ*_p-p_, as shown in Figure 15a. The changes of *V* for different *μ*_i-p_s and *μ*_w-p_s are similar, as shown in Figure 15b.

**Figure 16:**
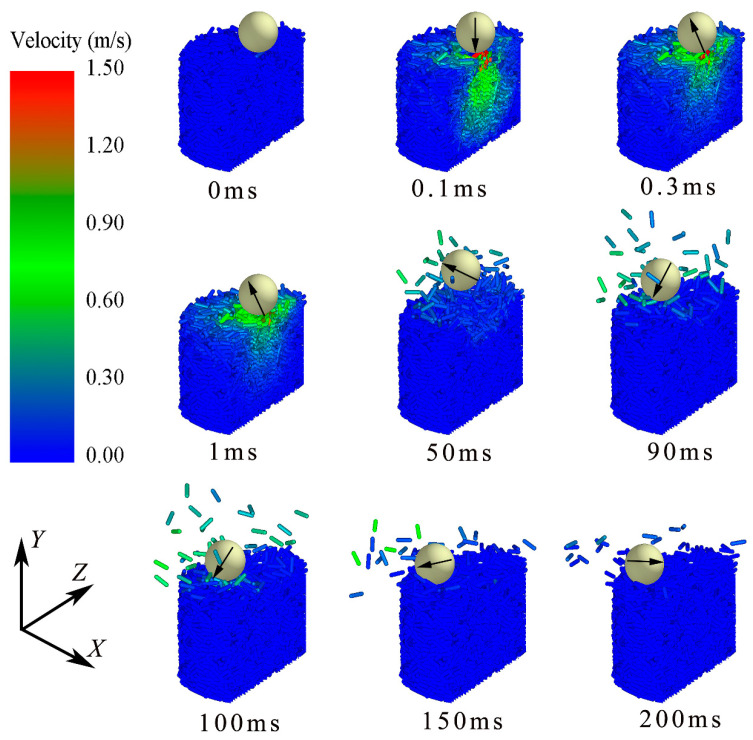
Velocity profiles of elongated particles (*L*_p_ = 6 mm) impacted by brass impactor. (*L*_p_ = 6 mm, *μ*_p-p_ = 0.8, arrow represents velocity of impactor, whose color remains unchanged.)

Figure 16 shows the velocity profiles of particles with *μ*_p-p_ = 0.8 at different times impacted by the brass impactor. The color of the granular bed represents the velocity magnitude, which is mainly in the range of 0.3 m/s–1 m/s. From Figure 14a and Figure 16, it can be found that the impactor rebounds to the height of 0.5*D*_0_ at *t* = 50 ms. Then, the impactor starts falling under gravity, and its velocity reaches a small peak at *t* = 90 ms, as shown in Figure 15a. Some ejected particles will contact with the impactor during *t* = 90–100 ms, as shown in Figure 16, which slows the impactor slightly. The second penetration begins at *t* = 90 ms, corresponding to the decreasing velocity, and the contact position is higher because the surface of the granular bed is irregular after the first impact. The maximum *H*_0_ is kept at 0.15*D*_0_ when *t* = 150 ms, indicating the termination of the whole penetration. As a whole, the velocity direction of the impactor is not always downward during its penetration, resulting in the spatial curve of the actual penetration path of the impactor. In addition, when the impactor rebounds, the trajectory of the impactor is a parabola, which makes the drop-point of the second penetration depart from that of the first one.

**Figure 17:**
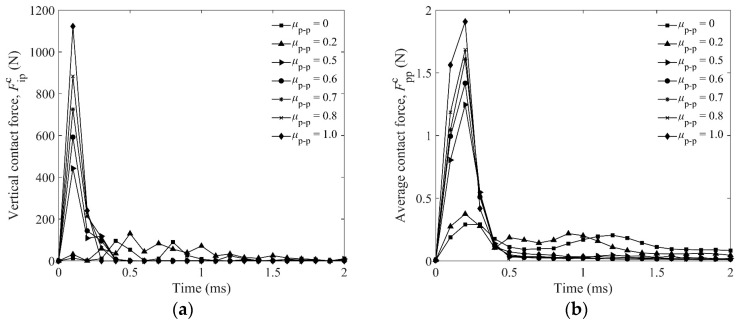
(**a**) $F_{\mathrm{ip}}^{c}$ as a function of time, $F_{\mathrm{ip}}^{c}$ is the vertical contact force between impactor and particles; and (**b**) $F_{\mathrm{pp}}^{c}$ as a function of time, $F_{\mathrm{pp}}^{c}$ is the average contact force between all particles.

Comparing the ejecta mass and penetration depth under different particle frictions (Figure 13 and Figure 14a), we presume that the objective particles would contact each other with a large contact force and build a strong quasi-continuous medium under a strong force chain to resist the penetration of the impactor, resulting in the decrease of *V*, as shown in Figure 15a. Therefore, we can say that the ejecta mass for a larger friction coefficient is decreased by the stronger force chain, because it takes more energy to separate the particles. Based on this consideration, we study the vertical contact force between the impactor and particles $F_{\mathrm{ip}}^{c}$, and the average contact force between particles and particles $F_{\mathrm{pp}}^{c}$, as shown in Figure 17a,b, respectively. It is obvious that the forces $F_{\mathrm{ip}}^{c}$ and $F_{\mathrm{pp}}^{c}$ increase with increasing *μ*_p-p_, and the force chain is the strongest when *μ*_p-p_ = 1.

### 4.3. Effect of Particle Configuration

**Figure 18:**
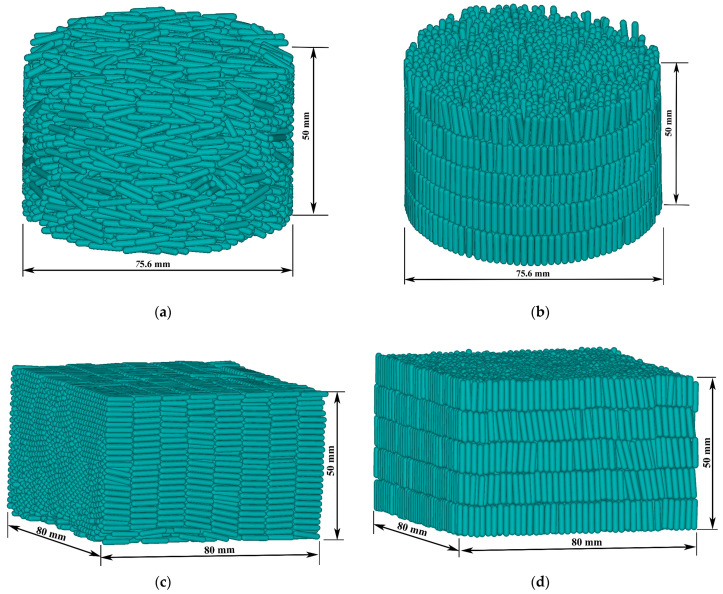
Diagram of regular configuration of elongated particles (*L*_p_ = 10 mm): (**a**,**b**) are lateral arrangement and vertical arrangement in the cylindrical cup; (**c**,**d**) are the lateral arrangement and vertical arrangement in the cuboid cup.

**Figure 19:**
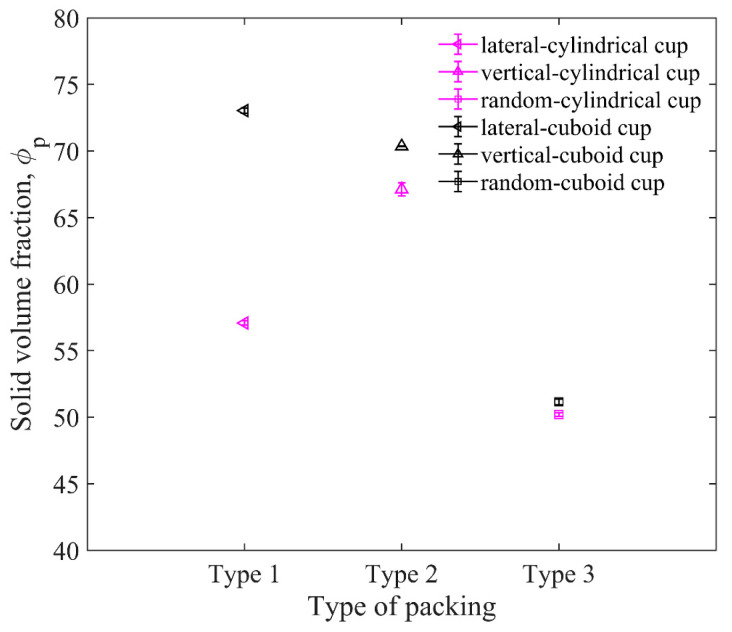
Comparison between solid volume fractions for different packing types (type 1—lateral arrangement, type 2—vertical arrangement, and type 3—random arrangement). The length of particles in the cup is 10 mm.

To find the effects of the shape of the cup and particle configuration, we chose steel elongated particles of *L*_p_ = 10 mm and the brass impactor to pack in the cylindrical and cuboid cup in the lateral and vertical directions, respectively, as shown in Figure 18. The friction coefficients and material parameters are shown in Table 2. In order to make the elongated particles more regular when they are arranged horizontally, the size of the cuboid cup is the integral multiple of the length of the elongated particles. The solid volume fractions *ϕ*_p_s are shown in Figure 19. It can be seen that the *ϕ*_p_s of the cuboid cup are larger than that of the cylindrical cup. The *ϕ*_p_ of vertical arrangement is the largest, and the *ϕ*_p_ of random packing is the smallest.

The orientational order of particles can be monitored by diagonalization of the symmetric traceless order tensor *Q* [38], as follows:

(12) $$Q_{\mathrm{ij}}=\frac{3}{2N}\sum_{n=1}^{N}l_{i}^{n}l_{j}^{n}-\frac{1}{3}\delta_{\mathrm{ij}}$$

where $l_{i\left(j\right)}^{n}$ is the unit vector along the major axis of the elongated particle n among all *N* particles. The largest eigenvalue of Q is the primary order parameter *S*_r_, which quantifies the degree of alignment. When *i* = *j*, $\delta_{\mathrm{ij}}$ = 1, or $\delta_{\mathrm{ij}}$ = 0, the order parameter *S*_r_ is equal to one if all particles are packed in the same direction, and *S*_r_ is zero if the orientation of every particle is different.

**Figure 20:**
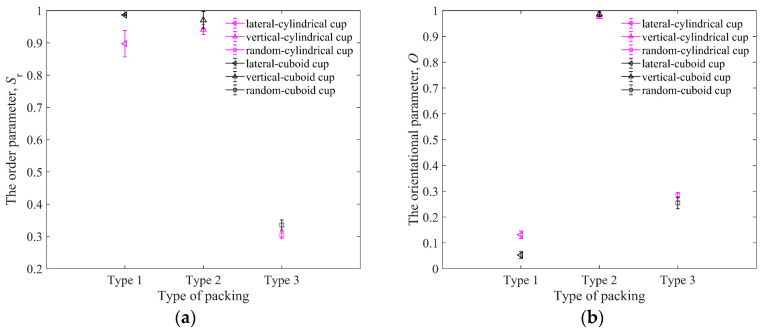
The order parameters and orientational parameters of three packings in cylindrical cup and cuboid cup: (**a**) is the order parameters for three packing types; (**b**) is the orientational parameters for three packing types (type 1—lateral arrangement, type 2—vertical arrangement, and type 3—random arrangement). The length of particles in the cup is *L*_p_ = 10 mm.

Although *S*_r_ can describe the order of particle configuration, it cannot show the direction of particle orientation. For example, if the major axes of all particles are all in the same inclined direction, the direction of particles cannot be specifically distinguished using Equation (12). Therefore, the average orientational parameter *O* of all particles is used as the quantitative analysis of the particle orientation. The orientational parameter [39] of a single elongated particle is calculated by the acute angle between the major axis of the elongated particle and the vertical axis. A larger *O* leads to a more vertical particle. Figure 20a,b show the order parameters *S*_r_ and orientational parameters *O* for three packing types in the cylindrical and cuboid cups. The values of *S*_r_ in the cuboid cup are all bigger than those in the cylindrical cup, which is caused by the restriction of the cylindrical wall.

**Figure 21:**
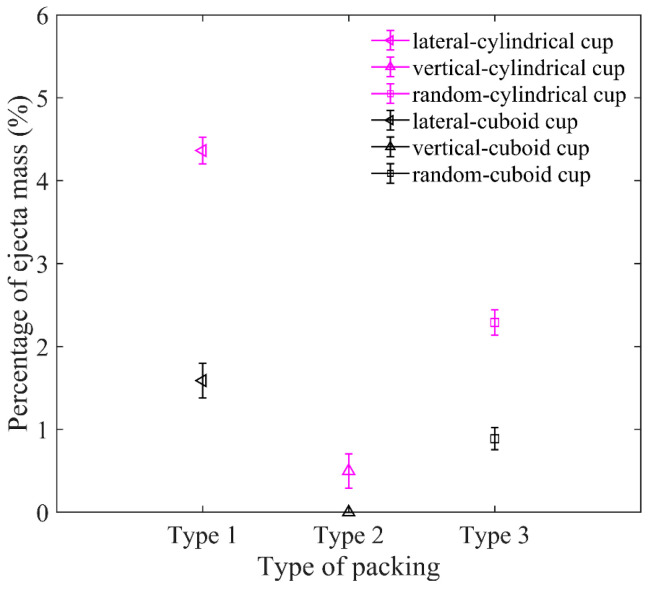
Comparison between ejecta masses for three packing types in cylindrical cup and cuboid cup. The length of particles in the cup is *AR* = 5.

**Figure 22:**
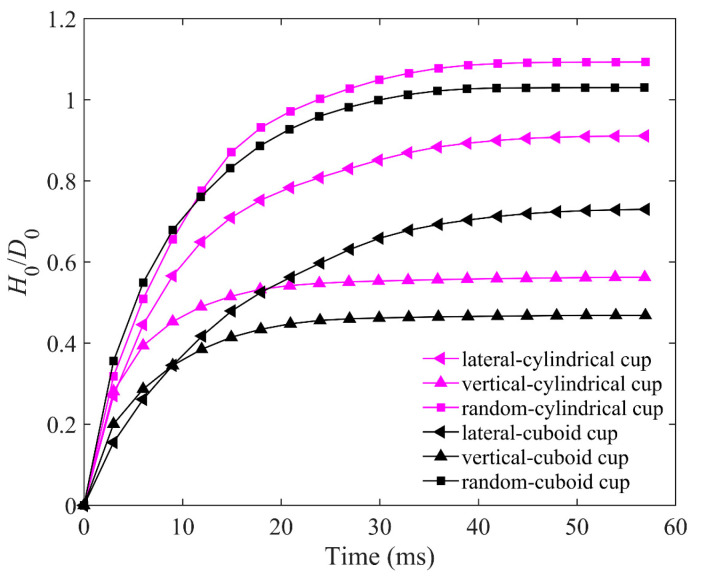
Comparison between *H*_0_/*D*_0_ for three packing types in cylindrical cup and cuboid cup. The length of particles in the cup is *AR* = 5; *H*_0_ and *D*_0_ represent the penetration depth of impactor and diameter of impactor, respectively.

As shown in Figure 21, the ejecta masses of the cylindrical cup are all larger than that of the cuboid cup. The ejecta mass of lateral arrangement is the largest, and that of vertical arrangement is the smallest. The elongated particles of lateral arrangement will easily roll out of the cup from the cup edge during impact, which leads to the larger ejecta masses of lateral arrangement. As shown in Figure 22, the *H*_0_ of the random packing is the largest, and the *H*_0_ of the vertical packing is the smallest in the two cups. In short, the cuboid cup has larger *S*_r_ and *ϕ*_p_, which lead to the smaller ejecta mass and penetration depth of the cuboid cup compared to those of the cylindrical cup. In addition, the vertical arrangement of elongated particles is more regular and has a stronger ability to resist penetration than the lateral arrangement.

## 5. Conclusions

Three-dimensional DEM simulations and experiments of spheres impacting into elongated particles at a low speed are investigated in this study. By validating the numerical simulations with the experimental results, we demonstrate the feasibility of using the above code to understand the sphere impacting elongated particles. Close agreement between the DEM simulations and experimental results can be obtained in terms of ejecta masses and initial solid volume fraction. The effects of the particle length, the friction, and the configuration of the elongated particle bed on impact are discussed in this study.

The effect of the sphere and elongated particles of four particles lengths on penetration depth, ejecta mass, granular temperature, and contact force are studied. Then, the friction coefficients *μ*_p-p_, *μ*_i-p_, and *μ*_w-p_ ranging from 0 to 1 are discussed; the impactor rebounds when *μ*_p-p_ is larger than 0.7. As for the configuration of the elongated particle bed, three packing types and two different cups are compared, ejecta mass of particles and penetration depth of the impactor are studied. Based on the present studies, the following conclusions can be drawn:

1. The effect of particle length. The ejecta mass of the spherical particle bed is obviously larger than that of the elongated particle bed. The granular bed of longer particles has a smaller penetration depth due to the spatial structure of elongated particles, although the solid volume fraction is smaller. In addition, the average contact force between particles is positively correlated to particle length. The average kinetic energy of the impactor obeys an exponential-like dissipation, and the particle length of the elongated particles has little effect on the energy allocation from the impactor to the ejected particles and particles in the cup.
2. The effect of friction. The *μ*_p-p_ has a significant effect on the ejecta mass and penetration depth of the impactor, while *μ*_i-p_ and *μ*_w-p_ have a limited effect. The ejecta mass and penetration depth are negatively correlated to *μ*_p-p_. The contact force between particles and particles or impactors are positively correlated to *μ*_p-p_.
3. The effect of particle configuration. The cuboid cup can obtain a more dense and regular granular bed. The ejecta mass and penetration depth of vertical arrangement are the smallest. For the same arrangement of elongated particles, the penetration depth is negatively correlated to order parameters and solid volume fraction.

## Figures and Tables

**Table 1:** Contact force models used in the DEM simulations.

	Scenarios	Models	
Group I	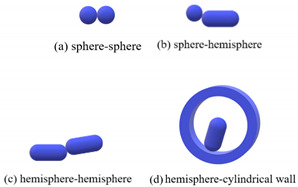	$F_{n}=\frac{4}{3}E^{*}R^{*}{}^{\frac{1}{2}}\delta_{n}^{\frac{3}{2}}$	(3)
		in which *δ*_n_ is the overlap in normal direction, E^*^ is equivalent Young’s modulus, and R^*^ is equivalent radius of two objects in contact, which are defined as $\frac{1}{E^{*}}=\frac{1-\nu_{1}^{2}}{E_{1}}+\frac{1-\nu_{2}^{2}}{E_{2}}$ and $\frac{1}{R^{*}}=\frac{1}{R_{1}}+\frac{1}{R_{2}}$, respectively, where *E*_1_ and *E*_2_, *R*_1_ and *R*_2_, *ν*_1_ and *ν*_2_ are the Young’s moduli, the radii, and the Poisson’s ratios of two contacting particles, respectively.	
Group II	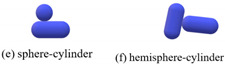	$F_{n}=8\sqrt{\frac{R^{*}}{27}}\alpha^{-\frac{3}{2}}E^{*}\delta_{n}^{\frac{3}{2}}$	(4)
		in which α is determined by the shape of contact area and determined to be 0.974 here.	
Group III	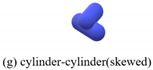	$F_{n}=\{\begin{array}{c} F_{n}^{\max}-\frac{10\left(F_{n}^{\max}-2F_{n}^{\min}\right)}{\pi }\theta ,\;\;\;0<\theta <\frac{\pi }{10}\\ 2F_{n}^{\min}-\frac{5F_{n}^{\min}}{2\pi }\left(\theta -0.1\pi \right),\;\;\;\frac{\pi }{10}\leq \theta <\frac{\pi }{2} \end{array}$	(5)
		in which *θ* is the angle between the two major axes of particles. $F_{n}^{\min}=\frac{4\sqrt{2}}{3}\kappa \sqrt{R^{*}}\alpha^{-\frac{3}{2}}E^{*}\delta_{n}^{\frac{3}{2}}$, when *θ* is equal to zero. $F_{n}^{\max}$ is the same as Equation (6).	
Group IV	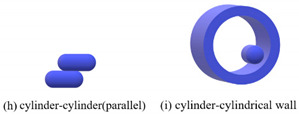	$F_{n}=\frac{\kappa \pi l}{1.8864+\ln\frac{l}{2b}}E^{*}\delta_{n}$	(6)
		in which *k* is a constant determined as 2.5, *l* is the length of contact area along the major axis, *b* is the width of contact area, $b=\sqrt{2R^{*}\delta_{n}}$.	
Tangential force model		$F_{t}=F_{t}^{0}+8G^{*}a\cdot v_{c}^{t}\cdot dt$	(7)
		where ***F***_t_^0^ and ***F***_t_ are the tangential force vectors in the previous time step and the current time step, respectively. G^*^ is governed by $\frac{1}{G^{*}}=\frac{2-\nu_{1}}{G_{1}}+\frac{2-\nu_{2}}{G_{2}}$, in which *G*_1_ and *G*_2_ are the shear moduli of the two objects in contact, *ν*_1_ and *ν*_2_ are the corresponding Poisson’s ratios. a is the effective radius of contact, $a=\sqrt{R^{*}\delta_{n}}$, and $v_{c}^{t}$d*t* represents the incremental tangential displacement in the present time step.	

Note: The equations are summarized according to the proposed models [36].

**Table 2:** Parameters used in the experiments and DEM simulations.

Parameters	Steel Objective Particles	Impactor	Granular Cup
diameter (mm)	*D*_p_ = 2.00	*D*_gls_ = 19.71/*D*_brs_ = 19.01	*D*_c_ = 75.60
height (mm)	*/*	/	*H*_c_ = 46.10
length (mm)	*L*_p_ = 4, 6, 8 *, 10 *	/	/
Young’s modulus (GPa) Poisson’s ratio	*E*_p_ = 182.00	*E*_gls_ = 71.70/*E*_brs_ = 117.00	*E*_c_ = 70.00
	*ν*_p_ = 0.30	*ν*_gls_ = 0.24/*ν*_brs_ = 0.34	*ν*_c_ = 0.35
density (kg/m^3^)	*ρ*_p_ = 8000.45	*ρ_gls_* = 2478.36/*ρ_brs_* = 8700.20	*ρ*_c_ = 2700.30
coefficient of friction	*μ*_p-p_ = **0.20**/0 ^#^	*μ*_i-p_ = **0.20**/0 ^#^	*μ*_w-p_ = **0.34**/0 ^#^
coefficient of restitution	*e*_p-p_ = 0.95	*e*_i-p_ = 0.88 (gls)/0.65(brs)	*e*_w-p_ = 0. 69
time step (s)	∆*t* = 2.30 × 10^−7^		
gravity (N/kg)	g = 9.81		
impact velocity (m/s)	*V*_0_ = 3.60		

Note: Values in bold font are the parameters used in base cases for simulations in this paper. The lengths marked with * are considered in DEM simulations only. The coefficients of friction marked with ^#^ are only used as the initial values in Section 4.2.

## Data Availability

No new data were created or analyzed in this study. Data sharing is not applicable to this article.

## Nomenclature

***F***_i_	external forces of particle *i* (N)
***T***_i_	external torques of particle *i* (N·m)
*m*_i_	mass of particle *i* (g)
***v***_i_	translational velocity of particle *i* (m/s)
*I*_i_	moment of inertia of particle *i* (kg·m^2^)
***ω***_i_	rotational velocity of the particle *i* (rad/s)
*δ*_n_	overlap in normal direction (mm)
*F*_n_	normal contact force (N)
*E^*^*	equivalent Young’s modulus
*R^*^*	equivalent radius of two objects
*F*_n_^min^	normal contact force when θ = 0° (N)
*F*_n_^max^	normal contact force when θ = 90° (N)
*l*	length of contact area along the major axis (mm)
*b*	width of contact area (mm)
***F***_t_	tangential contact force in the current time step (N)
***F***_t_^0^	tangential force vectors in the previous time step (N)
*G^*^*	equivalent shear modulus
*a*	effective radius of contact (mm)
$v_{c}^{t}$dt	incremental tangential displacement (mm)
$F_{n}^{d}$	normal damping force (N)
$F_{t}^{d}$	tangential damping force (N)
*m^*^*	equivalent mass
***v***_n_	normal component of relative velocity (m/s)
*v*_t_	tangential component of relative velocity (m/s)
*β*	contact damping coefficient
*S*_n_	normal contact stiffness
*S*_t_	tangential contact stiffness
*V*	vertical velocity of impactor (m/s)
*g*	acceleration of gravity (N/kg)
*m*_0_	total mass of particles in cup before impact (g)
*m*_1_	total mass of particles in cup after impact (g)
Δ*m*	mass of ejected particles (g)
*H*_0_	penetration depth of impactor (mm)
*D*_0_	diameter of impactor(mm)
*L*_p_	particle length (mm)
*E*_k0_	the initial kinetic energy of the impactor (J)
*E*_p0_	the initial potential energy of the impactor (J)
$E_{k}^{i}$	kinetic energy of the impactor (J)
$E_{k}^{c}$	kinetic energy of the particles in the cup (J)
$E_{k}^{e}$	kinetic energy of the ejected particles (J)
*T*_p_	granular temperature (m^2^/s^2^)
*ϕ*_p_	solid volume fraction
$F_{\mathrm{pp}}^{c}$	average contact force between particles (N)
*μ*_p-p_	coefficient of friction between target particles
*μ*_w-p_	coefficient of friction between wall and particles
*μ*_i-p_	coefficient of friction between impactor and particles
*T*_p,q_	granular temperature of particles along q (q = x, y, z) direction (m^2^/s^2^)
$F_{\mathrm{ip}}^{c}$	vertical contact force between impactor and particles (N)
*O*	orientational parameter
*S*_r_	the order parameter
